# Molecular epidemiology and clinical features of extended-spectrum beta-lactamase- or carbapenemase-producing *Escherichia coli* bacteremia in Japan

**DOI:** 10.1371/journal.pone.0202276

**Published:** 2018-08-29

**Authors:** Yuko Komatsu, Kei Kasahara, Takashi Inoue, Sang-Tae Lee, Tetsuro Muratani, Hisakazu Yano, Tadaaki Kirita, Keiichi Mikasa

**Affiliations:** 1 Center for Infectious Diseases, Nara Medical University, Nara, Japan; 2 Department of Oral and Maxillofacial Surgery, Nara Medical University, Nara, Japan; 3 Institute for Clinical and Translational Science, Nara Medical University, Nara, Japan; 4 Hibiki AMR Laboratory, Fukuoka, Japan; 5 Department of Clinical Laboratory, Kyurin Corporation, Fukuoka, Japan; 6 Department of Microbiology and Infectious Diseases, Nara Medical University, Nara, Japan; Hokkaido University Graduate School of Medicine, JAPAN

## Abstract

**Objectives:**

To identify risk factors and clinical outcomes in patients with bacteremia due to extended-spectrum beta-lactamase (ESBL) or carbapenemase-producing *Escherichia coli*, as well as to determine the prevalence and genetic background of such isolates.

**Methods:**

Case control study was performed with patients with *E*. *coli* bacteremia between January 2008 and May 2013 (n = 115) at a tertiary university hospital in Japan. Cases had ESBL-producing *E*. *coli* (ESBL-EC) whereas controls had non-ESBL-producing *E*. *coli* (non-ESBL-EC) isolates. A retrospective chart review was performed to identify risk factors and clinical outcomes. Isolates were characterized by antimicrobial susceptibility testing, polymerase chain reaction analysis for beta-lactamase genes, and multi-locus sequence typing.

**Results:**

Of 115 unique cases of *E*. *coli* bacteremia, 30 (26.1%) were due to ESBL-EC and three (2.6%) were due to carbapenemase-producing *E*. *coli*. All three carbapenemase-producing *E*. *coli* isolates were IMP-6 and concurrently produced ESBL (ESBL/IMP-6-EC). ESBL-EC isolates showed multidrug resistance. Of the ESBL-EC isolates, CTX-M-27 was the most prevalent (33.3%), followed by CTX-M-14 (30%). Multi-locus sequence typing revealed that 19 (63.3%) isolates were ST131. The multivariate analysis identified nursing home-associated infections and antibiotic administration in the preceding 30 days as risk factors for ESBL-EC bacteremia. The 14-day mortality non-ESBL-EC, ESBL-EC, and ESBL/IMP-6-EC was 4.7% (4/85), 20% (6/30), and 66.7% (2/3), respectively.

**Conclusions:**

CTX-M-27, CTX-M-14, and ST131 were the most prevalent ESBL-EC isolates from bacteremic patients in a Japanese hospital. Further studies with larger sample sizes are warranted to investigate the clinical significance of ESBL-EC and ESBL/IMP-6-EC.

## Introduction

*Escherichia coli* is a common commensal organism in the intestinal tracts of humans and animals; it causes a wide range of diseases. Resistance against various antimicrobials, including the cephalosporins, fluoroquinolones, and even the carbapenems, is increasing worldwide [1]. The genetic background of such resistance has been extensively studied and varies according to geographic location and time. The *E*. *coli* sequence type (ST) 131 C2/H30Rx clade with the *bla*_CTX-M-15_ gene is largely responsible for the global dissemination of extended-spectrum beta-lactamase-producing *E*. *coli* (ESBL-EC) [2]. In Japan, Matsumura et al. evaluated cases of bacteremia due to ESBL-EC between 2005 and 2010 and found the *bla*_CTX-M-14_ gene to be the most common, followed by *bla*_CTX-M-15_ and *bla*_CTX-M-2_ [3]. They recently reported on the global emergence and increased prevalence of the *E*. *coli* ST131 clade with the *bla*_CTX-M-27_ gene, named the C1-M27 clade [4]. Class A carbapenemases such as *Klebsiella pneumoniae* carbapenemase (KPC), and class B metallo-beta-lactamases such as the New Delhi Metallo-beta-lactamase (NDM), are a problem worldwide; however, these are rarely found in Japan where other types of metallo-beta-lactamases, such as IMP-6, are dominant [5, 6]. Although the incidence is low, Yamamoto et al. recently reported that 14.9% of long-term hospitalized patients harbored carbapenem-resistant Enterobacteriaceae, 95.7% of which produced IMP-6 [7–9]. The clinical impact of IMP-6-producing organisms has not yet been reported.

In this study, we aimed to identify the prevalence and genes associated with ESBL-EC and carbapenemase-producing *E*. *coli*, as well as to identify the risk factors and outcomes of patients with bacteremia caused by these organisms.

## Materials and methods

### Study setting and study design

This study was conducted at Nara Medical University, a tertiary care hospital with 927 beds in Nara prefecture, located in central Japan. All patients aged 18 years or older with at least one positive blood culture for *E*. *coli* between January 1, 2008, and May 31, 2013, were identified via the clinical microbiology laboratory’s computerized database. A case-control study design was used to determine risk factors for the ESBL-EC bacteremia group. The case group comprised patients with ESBL-EC bacteremia and the control group comprised patients with non-ESBL-EC bacteremia. Only the first episode of bacteremia was included for each patient. Ethical approval was obtained from the Institutional Review Board of Nara Medical University (No. 802).

### Microbiological analysis

The clinical microbiology laboratory used the BacT/Alert 3D blood culture system (Sysmex bioMérieux, Tokyo, Japan) and identification of bacterial isolates was performed using the VITEK^®^ 2 system (Sysmex bioMérieux, Tokyo, Japan). The minimum inhibitory concentration of various antimicrobial agents was determined using the agar dilution method and was interpreted according to the Clinical and Laboratory Standards Institute’s (CLSI) guidelines [10]. Isolates were reported as being susceptible to flomoxef at a minimum inhibitory concentration of ≤ 8 μg/mL, in reference to the CLSI breakpoint for moxalactam (≤ 8 μg/mL). Screening for ESBL production was performed using the VITEK^®^ 2 Advanced Expert System according to the manufacturer instructions; ESBL production was confirmed using the combined disk test according to CLSI guidelines [10]. Bacterial DNA was isolated using the QIAamp DNA Mini kit (Qiagen, Hilden, Germany). Polymerase chain reaction analyses for the detection of TEM-, SHV-, CTX-M-type beta-lactamase genes, and plasmid-mediated AmpC beta-lactamases (p-AmpC) were performed as previously described [11, 12]. Isolates displaying non-susceptibility to imipenem or meropenem (minimum inhibitory concentration > 1 μg/mL) were analyzed to determine the presence of carbapenemases, using primers as previously described [6].

### Clinical analysis

Bacteremia was categorized as nosocomial, healthcare-associated, or community acquired, in accordance with the criteria set out by Friedman et al Nursing home-associated infections comprised those occurring in patients residing in nursing homes or who attended day care within 30 days of the onset of bacteremia [13]. The Charlson comorbidity index was used to categorize comorbid conditions, identified by reviewing the patients’ medical charts [14]. Other clinical information included age; sex; date of onset of nosocomial infection; whether antibiotic agents, general anesthesia, chemotherapy, radiation therapy, or immunosuppressive agents such as glucocorticoids were administered within 30 days of the date of onset of bacteremia; intensive care unit admission at the time of bacteremia; source of infection (urinary tract, intra-abdominal, catheter-associated, soft-tissue, pneumonia, or unknown); presence of indwelling devices (peripheral or central venous catheters, urinary catheter, drainage tube(s), nasogastric tube, tracheotomy tube, and devices related to oxygen inhalation, mechanical ventilation, or continuous hemodiafiltration). Inappropriate antimicrobial treatment was defined as the use of an antimicrobial agent to which the pathogen being treated is resistant.

### Statistical analysis

The statistical analysis was performed using Stata software version 13 (Stata Corporation College Station, TX, USA). For the univariate statistical analysis of dichotomous outcomes, Fisher’s exact test and logistic regression analysis were used to compare categorical and continuous explanatory variables, respectively. Odds ratios (ORs) and 95% confidence intervals (CIs) were calculated to determine the strength of any associations that emerged. P values < .05 were considered statistically significant, and all probabilities were two-tailed. In the risk factor analysis for ESBL-EC bacteremia, multivariate logistic regression analysis was performed with nosocomial infection, nursing home-associated infection, and used antibiotic(s) within 30 days. It was thought that nosocomial and nursing home-associated infection might be associated with ESBL-producing organism acquisition while the last factor (used antibiotic(s) within 30 days) may be associated with ESBL-producing organisms’ selection. These factors have been shown to be clinically significant variables of ESBL-producing organisms in previous studies [15–17]. The number of variables included in the multivariate analysis was restricted to 10% of the number of ESBL-producing isolates [18]. In the risk factor analysis for 14-day mortality, a multivariate analysis could not be performed because there were only ten deaths.

## Results

### Microbiology results

During the study period, there were 115 unique cases of bacteremia caused by *E*. *coli*: 30 (26.1%) were due to ESBL-EC and 85 (73.9%) were due to non-ESBL-EC. The antibiotic susceptibility patterns of all isolates are shown in Table 1. Among the cases, a high proportion was resistant to piperacillin-tazobactam, cefmetazole, gentamicin, tobramycin, levofloxacin, ciprofloxacin, and trimethoprim-sulfamethoxazole. Notably, 83.3% of cases were resistant to ciprofloxacin and 50% were resistant to trimethoprim-sulfamethoxazole, compared with 15.3% and 17.6% of controls, respectively.

**Table 1:** Antimicrobial resistance of ESBL-EC and non-ESBL-EC isolates.

Antimicrobial agent	ESBL-EC (n = 30)	Non-ESBL-EC (n = 85)	*P* value
Piperacillin-tazobactam	5 (16.7)	1 (1.2)	.005
Cefmetazole	5 (16.7)	2 (2.4)	.01
Flomoxef	1 (3.3)	1 (1.2)	.46
Imipenem	1 (3.3)	0	.26
Meropenem	1 (3.3)	0	.26
Gentamicin	12 (40)	5 (5.9)	< .001
Tobramycin	12 (40)	5 (5.9)	< .001
Amikacin	1 (3.3)	0	.26
Levofloxacin	24 (80)	14 (16.5)	< .001
Ciprofloxacin	25 (83.3)	13 (15.3)	< .001
Fosfomycin*	0	3 (3.5)	.57
Trimethoprim-sulfamethoxazole	15 (50)	15 (17.6)	< .001

Data are presented as n (%). ESBL-EC, extended-spectrum beta-lactamase-producing *E*. *coli*; non-ESBL-EC, non-extended-spectrum beta-lactamase-producing *E*. *coli*.

* Clinical Laboratory Standards Institute breakpoint is available for urine isolates only.

The ST and distribution of antimicrobial resistance genes of the ESBL-EC isolates are shown in Table 2. CTX-M-27 was most prevalent, followed by CTX-M-14. Three isolates possessed both CTX-M (two CTX-M-2 and one CTX-M-27) and IMP-6 genes. ST131 was the most prevalent ST (19/30, 63.3%). CTX-M-27 (8/19, 42.1%) and CTX-M-14 (5/19, 26.3%) were the most common ESBL-associated genes. Two of the three CTX-M+IMP-6 isolates were ST131.

**Table 2:** Distribution of ESBL-EC antimicrobial resistance genes and sequence types.

Genotype (n = 30 cases)	Number (%) of isolates	MLST (number of isolates)
CTX-M-27	10 (33.3)	ST131 (8), ST2179 (1), ST2750 (1)
CTX-M-14	7 (23.3)	ST131 (5), ST62 (1), ST405 (1)
CTX-M-2 &IMP-6	2 (6.7)	ST131 (1), ST2750 (1)
CTX-M-2	2 (6.7)	ST131 (2)
CTX-M-14 & CMY-2	2 (6.7)	ST95 (1), ST648 (1)
SHV-12	1 (3.3)	ST362 (1)
Novel CTX-M^a^	1 (3.3)	ST648 (1)
CTX-M-61	1 (3.3)	ST131 (1)
CTX-M-27 & IPM-6	1 (3.3)	ST131 (1)
CTX-M-19	1 (3.3)	ST131 (1)
CTX-M-15	2 (3.3)	ST847 (1), ST2179 (1)

MLST, multi-locus sequence typing; ESBL-EC, extended-spectrum beta-lactamase producing *E*. *coli*.

^a^ GenBank accession number: KY964289

### Clinical results

The patients’ demographic characteristics and risk factors for ESBL-EC bacteremia are listed in Table 3. The following variables were identified as risk factors for ESBL-EC bacteremia in the univariable analysis: younger age; community-onset, nosocomial, and nursing home-associated infections; administration of antibiotic, general anesthetic, or immunosuppressive agents within 30 days of bacteremia onset; connective tissue disease, paraplegia or hemiplegia, dementia, and leukemia; a higher Charlson comorbidity index; and urinary or central venous catheterization. In the multivariate analysis, nursing home-associated infection (OR, 7.98; 95% CI, 1.56–40.9) and antibiotic administration within the preceding 30 days (OR, 5.11; 95% CI, 1.61–16.2) were independent risk factors for ESBL-EC bacteremia; however nosocomial infection (OR, 1.28; 95% CI, 0.4–4.17) was not. We quantified the severity of multicollinearity among the predictor variables in the regression analysis by calculating the variance inflation factor (VIF). The VIF values were 1.48, 1.05, and 1.44 for nosocomial and nursing home-associated infections, and used antibiotics within 30 days, respectively. The fact that these variables were sufficiently smaller than 5.0 means that each predictor variable was uncorrelated with the other predictors and the multicollinearity among them was vanishingly small.

**Table 3:** Univariate and multivariate analysis of risk factors associated with ESBL-EC compared with non-ESBL-EC isolates in bacteremic patients.

Variable	ESBL-EC	Non-ESBL-EC	Univariate analysis		Multivariate analysis	
	(n = 30)	(n = 85)	OR (95% CI)	*P* value	OR (95% CI)	*P* value
Age, mean (SD), y	66.5 (17.2)	72.3 (10.8)	0.97 (0.94–1.00)	.04		
Sex: male	19	37	2.22 (0.88–5.87)	.09		
Healthcare-associated infection	9	31	0.75 (0.27–1.97)	.66		
Nosocomial infection	21	40	2.63 (1.00–7.25)	.04	1.28 (0.40–4.17)	.678
Nursing home-associated infection	6	3	6.69 (1.31–44.50)	.009	7.98 (1.56–40.9)	.013
Days to onset of nosocomial infection, median (IQR)	24 (15–79)	28 (12–50)	1.00 (0.99–1.01)	.63		
Used antibiotic(s) within 30 days	22	28	5.51 (2.05–16.22)	< .001	5.11 (1.61–16.2)	.006
General anesthesia within 30 days	7	6	3.94 (1.02–15.8)	.04		
Chemotherapy within 30 days	2	11	0.48 (0.05–2.43)	.51		
Radiation within 30 days	1	3	0.94 (0.02–12.29)	>.99		
Immunosuppressants within 30 days	14	21	2.64 (1.01–6.93)	.04		
Intensive care unit admission before infection	8	10	2.70 (0.82–8.70)	.08		
Source of infection:						
Urinary tract	13	49	0.56 (0.22–1.41)	.20		
Intra-abdominal	10	22	1.43 (0.51–3.81)	.48		
Catheter-associated	2	2	2.93 (0.20–42.2)	.28		
Soft-tissue	1	1	2.86 (0.04–229.6)	.46		
Pneumonia	0	4	0 (0–4.32)	.57		
Unknown	4	7	1.71 (0.34–7.37)	.47		
Comorbid condition(s):						
Malignancy	11	38	0.72 (0.27–1.82)	.52		
Diabetes mellitus	8	19	1.26 (0.42–3.56)	.63		
Connective tissue disease	7	3	8.12 (1.69–52.53)	.003		
Cerebral vascular disease	5	9	1.68 (0.40–6.23)	.52		
Peripheral vascular disease	3	4	2.23 (0.31–14.12)	.37		
Paraplegia or hemiplegia	6	2	10.11 (1.67–108.70)	.004		
Dementia	6	1	20.3 (2.30–971.50)	.001		
Cardiovascular disease	5	15	0.93 (0.24–3.08)	>.99		
Chronic liver disease	4	13	0.85 (0.19–3.10)	>.99		
Renal insufficiency	4	13	0.85 (0.19–3.10)	>.99		
Chronic pulmonary disease	5	8	1.91 (0.45–7.37)	.32		
Leukemia	4	0	Inf (1.99–Inf)	.004		
Lymphoma	2	2	2.93 (0.20–42.19)	.28		
Peptic ulcer	2	0	Inf (0.54–Inf)	.07		
Charlson comorbidity index, mean (SD)	4.2 (2.2)	2.7 (2.1)	1.34 (1.11–1.62)	.003		
Charlson comorbidity index >2	21	33	3.63 (1.39–10.18)	.005		
Indwelling devices:						
Peripheral catheterization	10	17	1.99 (0.70–5.50)	.21		
Urinary catheterization	10	11	3.32 (1.10–10.07)	.03		
Oxygen inhalation	7	11	2.05 (0.60–6.56)	.24		
Central venous catheter	7	4	6.04 (1.39–30.70)	.007		
Drainage tube	2	6	0.94 (0.09–5.66)	>.99		
Tracheotomy tube	2	2	2.93 (0.20–42.19)	.28		
Nasogastric tube	2	2	2.93 (0.20–42.19)	.28		
Mechanical ventilation	2	2	2.963(0.20–42.19)	.28		
Continuous hemodiafiltration	1	1	2.86 (0.04–229.6)	.46		

ESBL-EC, extended-spectrum beta-lactamase-producing *E*. *coli*; non-ESBL-EC, non-extended-spectrum beta-lactamase-producing *E*. *coli*; OR, odds ratio; CI, confidential interval; IQR, interquartile range; SD, standard deviation; CCI, Charlson comorbidity index; Inf, infinity.

Overall, 14 day-mortality was 8.7% (10/115) with 4.7% (4/85) for non-ESBL-EC, 14.8% (4/27) for ESBL-only-EC, 20% (6/30) for ESBL-EC, and 66.7% (2/3) for ESBL/IMP-6-EC. Table 4 shows the results of the univariate analysis of 14-day mortality. Multiple factors, such as the use of inadequate antibiotic therapy within 24 hours; prior use of any antibiotic and specifically of any carbapenem; infection arising from the urinary tract, soft-tissue, or an unknown source; chronic liver disease; central venous catheterization; presence of a nasogastric tube; mechanical ventilation; ESBL-EC and ESBL/IMP-6-EC were also significantly associated with 14-day mortality.

**Table 4:** Univariate analysis of risk factors for all-cause mortality within 14 days after onset of bacteremia due to *E*. *coli*.

Variables	Survived (n = 105)	Died (n = 10)	OR	95% CI	*P* value
Sex: male	50	6	1.53	0.43–5.41	.51
Intensive care unit after infection	17	4	2.91	0.82–10.33	.08
Inadequate antibiotic therapy within 24 h	9	5	9.11	2.63–31.55	< .001
Community-onset infection	51	3	0.55	0.14–2.15	.38
Healthcare-associated infection	38	2	0.53	0.11–2.48	.41
Nosocomial infection	54	7	1.80	0.47–6.99	.38
Nursing home-associated infection	7	2	2.80	0.59–13.19	.17
Antibiotic(s) used within 30 days:					
Any antibiotic	41	9	10.93	1.38–86.3	.004
Any carbapenem	11	5	6.18	1.79–21.4	< .001
Any quinolone	7	1	1.52	0.19–12.0	.69
Immunosuppressant used within 30 days	30	5	2.18	0.63–7.52	.21
General anesthesia within 30 days	12	1	0.79	0.10–6.22	.82
Chemotherapy within 30 days	11	2	1.84	0.39–8.68	.43
Radiation within 30 days	4	0	<0.001	0–Inf	.52
Intensive care unit admission	16	2	1.21	0.26–5.68	.81
Source of infection:					
Urinary tract	60	2	0.21	0.046–1.01	.03
Intra-abdominal	28	4	1.71	0.48–6.05	.40
Catheter-associated	4	0	<0.001	0–Inf	.52
Soft-tissue	1	1	8.21	1.03–65.3	.02
Pneumonia	4	0	<0.001	0–Inf	.52
Unknown	8	3	4.39	1.13–17.0	.02
Comorbid condition(s):					
Malignancy	45	4	0.86	0.24–3.04	.81
Diabetes mellitus	25	2	0.83	0.18–3.9	.81
Connective tissue disease	9	1	1.07	0.14–8.45	.95
Cerebrovascular disease	12	2	1.95	0.41–9.19	.39
Peripheral vascular disease	7	0	<0.001	0–Inf	.39
Paraplegia or hemiplegia	7	1	1.20	0.43–3.36	.73
Dementia	7	0	<0.001	0–Inf	.39
Cardiovascular disease	19	1	0.54	0.068–4.26	.55
Chronic liver disease	12	5	6.26	1.81–21.7	< .001
Renal insufficiency	15	2	1.46	0.31–6.86	.63
Chronic pulmonary disease	11	2	2.10	0.45–9.89	.34
Leukemia	3	1	3.55	0.45–28.0	.20
Lymphoma	4	0	<0.001	0–Inf	.52
Peptic ulcer	2	0	<0.001	0–Inf	.65
Indwelling device:					
Peripheral catheter	25	2	0.74	0.16–3.50	.70
Urinary catheter	17	4	2.78	0.78–9.85	.10
Oxygen inhalation	15	3	2.15	0.56–8.31	.25
Central venous catheter	8	3	4.18	1.08–16.18	.02
Drainage tube	6	2	3.16	0.67–14.87	.12
Tracheotomy tube	2	2	6.50	1.38–30.61	.006
Nasogastric tube	1	3	13.17	3.39–51.14	< .001
Mechanical ventilation	2	2	6.50	1.38–30.61	< .001
Continuous hemodiafiltration	1	1	5.54	0.70–43.7	.07
Empiric treatment with a carbapenem	43	6	1.89	0.53–6.70	.31
Carbapenem use after susceptibility test	66	4	0.36	0.10–1.27	.09
Carbapenem use	68	6	0.71	0.20–2.51	.59
Age ≥ 65 years	85	6	0.36	0.10–1.28	.10
Charlson comorbidity score of > 2	47	7	2.62	0.68–10.12	.15
Age, mean years (SD)	71.4 (12.1)	64.1 (19.5)	0.97	0.93–1.01	.10
Charlson comorbidity index, mean (SD)	4.3 (3.1)	3.0 (2.1)	1.26	0.97–1.62	.08
ESBL-only-EC	23	4	2.36	0.45–10.94	.24
ESBL-EC	24	6	4.97	1.08–26.01	.02
ESBL/IMP-6-EC	1	2	24.17	1.15–1527.7	.02

OR, odds ratio; CI, confidential interval; SD, standard deviation; ESBL-only-EC, extended-spectrum beta-lactamase-producing only *E*. *coli*; ESBL-EC, extended-spectrum beta-lactamase-producing *E*. *coli*; non-ESBL-EC, non-extended-spectrum beta-lactamase-producing *E*. *coli*

## Discussion

Worldwide, the prevalence of ESBL-EC has varied widely and is increasing. Matsumura et al. found that 13.9% of 706 cases of *E*. *coli* bacteremia occurring from 2005–2012 were due to ESBL-producing strains, while Namikawa et al. recently reported that 24.0% of 129 cases of *E*. *coli* bacteremia from 2011–2015 were due to ESBL-EC [3, 19]. In our study, a total of 115 unique cases of *E*. *coli* bacteremia were identified and, of these, 30 (26.1%) were caused by ESBL-EC. The proportion of carbapenemase-producing *E*. *coli* was 2.6%, and all cases produced IMP-6. The prevalence of carbapenemase-producing *E*. *coli* among all cases of *E*. *coli* bacteremia in Japan has not previously been reported. In a recent analysis of 4875 Enterobacteriaceae isolates collected in Japan between 2010 and 2013, Ohno et al. reported that the prevalence of IMP-6-producing strains ranged from 0.08% to 0.92%, depending on the species and biologic specimen from which the isolates were cultured [20]. Because IMP-6-producing isolates typically exhibit low-level resistance to the carbapenems, they are often overlooked; however, it is important that these strains be continuously monitored using highly sensitive tests [6].

Multilocus sequence typing revealed that 19 of the 30 ESBL-EC isolates (63.3%) were ST131. The global increase in ESBL-EC is associated with a pandemic clonal group known as ST131 that includes CTX-M-type-producing ESBLs [6]. It is known that ST131 *E*. *coli* sequentially acquires antimicrobial resistance genes and develops resistance to multiple classes of antibiotics, including aminoglycosides, fluoroquinolones, and sulfamethoxazole-trimethoprim [2]. This could explain the high rates of multi-class antibiotic resistance among the ESBL-EC isolates in our study. Matsumura et al. recently reported an increase in CTX-M-27-producing ST131 isolates in Japan, named C1-M27 [4]. A third of our ESBL isolates produced CTX-M-27, and 80% of these were ST131. The second most prevalent CTX-M was CTX-M-14, 71% of which were ST131. Of 115 isolates, three (2.6%) produced both IMP-6 and either CTX-M-2 or CTX-M-27; and two were ST131. *E*. *coli* strains producing both IMP-6 and CTX-M-2 have been reported, although this is the first study to report an *E*. *coli* isolate producing both IMP-6 and CTX-M-27 [21]. This finding alerts us to the future risk that C1-M27 might acquire higher-level antimicrobial resistance genes, such as carbapenemase-resistance genes.

As reported, many factors are associated with infection or colonization by ESBL-EC [6, 7, 9, 10, 19, 20, 22–24]. By comparing ESBL-EC bacteremia and non ESBL-EC bacteremia, we identified risk factors of ESBL-EC in patients with EC bacteremia. At univariate analysis, multiple factors were found to be associated with ESBL-EC; however, at multivariate analysis, we chose three representative risk factors due to sample size constraints. These three factors were traditionally regarded as being important for the isolation of multidrug resistant organisms [15–17]. Historically, multidrug resistant organisms are isolated from severely ill, hospitalized patients in intensive care [23]. However, community acquired ESBL-EC infection is reported to be as important as nosocomial ESBL-EC infection [25]. From this perspective, it is interesting that nosocomial infection was not a significant risk factor for ESBL-EC bacteremia at multivariate analysis. On the other hand, it has been reported that residents in nursing homes have high risk of carriage of ESBL-EC [24, 26]. Our result is consistent with these reports in that nursing home-associated infection was significantly associated with ESBL-EC bacteremia. Finally, exposure to antibiotics has been reported as the most significant risk factor in many studies [1, 5, 9, 15–17, 19, 24, 26]. Antibiotic exposure is associated with multidrug resistant organisms, not only by inducing mutations associated with antibiotic resistance, but also by selecting resistant organisms; which is called selection pressure. In our study, antibiotic exposure within 30 days was a significant risk factor for ESBL-EC bacteremia.

A number of studies found no significant association between ESBL production and crude mortality [15–17, 19]. Conversely, several other studies observed that patients with infection due to antibiotic-resistant organisms tended to have poorer outcomes [22, 27]. Inappropriate empiric treatment, frequently observed in antibiotic-resistant *E*. *coli* infection, is the main determinant of mortality. Again, due to sample size constraints, we performed only univariate analysis, revealing multiple significant risk factors of death. Patients with ESBL-EC bacteremia and ESBL/IMP-6-EC bacteremia may have poorer prognosis; however, there is need for further study to appropriately identify the risk factors of death.

There are some limitations to our study. First, it involved a single center; hence, the generalizability of our results to other settings may not be feasible. Second, the limited number of patients prevented us from performing a more detailed statistical analysis. The difference in mortality among non-ESBL-EC, ESBL-EC, and ESBL/IMP-6-EC cannot be attributed to the presence of resistance. For the aim to identify clinically relevant risk factors of resistance and mortality, a multicenter study with a larger study population is warranted.

In conclusion, of 115 unique bloodstream infections caused by *E*. *coli*, 30 (26.1%) were caused by ESBL-EC and three (2.6%) by carbapenemase-producing *E*. *coli*. All carbapenemase-producing *E*. *coli* were IMP-6-producing strains that also produced ESBLs. Among the ESBL-EC, 33.3% possessed CTX-M-27 and 30% harbored CTX-M-14. Nineteen (63.3%) were ST131. Nursing home-associated infections and administration of antibiotic agents within the previous 30 days were associated with ESBL-EC bacteremia. Overall, 14 day-mortality was 8.7% (10/115) with 4.7% (4/85) for non-ESBL-EC, 14.8% (4/27) for ESBL-only-EC, 20% (6/30) for ESBL-EC, and 66.7% (2/3) for ESBL/IMP-6-EC; however, the sample size in this study was not sufficient to evaluate the clinical impact on mortality, and further studies with larger sample sizes are warranted.
